# Effect of Dietary Supplements in Reducing Probability of Death for Uremic Crises in Dogs Affected by Chronic Kidney Disease (Masked RCCT)

**DOI:** 10.1100/2012/219082

**Published:** 2012-04-19

**Authors:** Andrea Zatelli, Marco Pierantozzi, Paola D'Ippolito, Mauro Bigliati, Eric Zini

**Affiliations:** ^1^Clinica Veterinaria Pirani, Nephrology and Urology Division, Via Majakowski 2/L,M,N, 42124 Reggio Emilia, Italy; ^2^Istituto Farmaceutico Candioli, Via Manzoni 2, 10192 Beinasco, Italy; ^3^Istituto Veterinario di Novara, S.P. 9, 28060 Granozzo con Monticello, Italy; ^4^Department of Veterinary Clinical Sciences, University of Padua, 35020 Agripolis, Legnaro, Italy

## Abstract

Chitosan and alkalinizing agents can decrease morbidity and mortality in humans with chronic kidney disease (CKD). Whether this holds true in dog is not known. Objective of the study was to determine whether a commercial dietary supplement containing chitosan, phosphate binders, and alkalinizing agents (Renal), compared to placebo, reduces mortality rate due to uremic crises in dogs with spontaneous CKD, fed a renal diet (RD). A masked RCCT was performed including 31 azotemic dogs with spontaneous CKD. Dogs enrolled in the study were randomly allocated to receive RD plus placebo (group A; 15 dogs) or RD plus Renal (group B; 16 dogs). During a first 4-week period, all dogs were fed an RD and then randomized and clinically evaluated up to 44 weeks. The effects of dietary supplements on mortality rate due to uremic crises were assessed. At 44 weeks, compared to group A, dogs in group B had approximately 50% lower mortality rate due to uremic crises (*P* = 0.015). Dietary supplementation with chitosan, phosphate binders, and alkalinizing agents, along with an RD, is beneficial in reducing mortality rate in dogs with spontaneous CKD.

## 1. Introduction

There is a strong consensus to use dietary modification in dogs affected by chronic kidney disease (CKD) [1–7]. Feeding a renal diet (RD) in dogs with mild and moderate spontaneous CKD had beneficial effects on uremia and mortality rate compared to a maintenance diet [1]. In addition, phosphate retention and renal secondary hyperparathyroidism are common complications of CKD [2–7], and hyperphosphatemia is associated with the development of renal lesions in dogs and cats [2–7]. In humans and cats, oral supplementation with compounds such as chitosan (produced by deacetylation of chitin, which is the structural element in the exoskeleton of crustaceans and cell wall of fungi), calcium carbonate, and potassium citrate has been advocated to control hyperphosphatemia [4–11]. In addition, chitosan is recognized to reduce azotemia during spontaneous CKD in humans and cats [8–10]. However, whether this holds true for dog has not been previously assessed. Aim of the present study was to evaluate the efficacy of a commercial oral supplement rich in chitosan, enteric phosphate binders, and alkalinizing agents (Renal—Istituto Farmaceutico Candioli SpA, Italy), in reducing mortality rate due to uremic crises in dogs affected by spontaneous CKD, in International Renal Interest Society (IRIS) stages 2, 3, and 4 [4, 12], fed an RD.

## 2. Materials and Methods

### 2.1. Animals

Dogs affected by CKD, in IRIS stages 2, 3, and 4, were recruited at the Clinica Veterinaria Pirani of Reggio Emilia, Italy. Results of history, physical examination, including body weight (BW) and body condition score (BCS) (1 to 5 scoring system (3 optimal)), CBC, serum biochemical profile, urinalysis, urine protein-to-creatinine (UPC) ratio, venous blood gas analysis, and indirect blood pressure measurement were collected. All dogs underwent abdominal ultrasonographic examination, which was performed by the same operator and with the same instrument (Philips HD11XE or Philips HD7XE, Philips Ultrasound, Bothell, Washington, USA). Dogs of any age were included if presenting inactive urine sediment and stable renal function, as defined by serum creatinine concentrations above 1.4 mg/dL (IRIS stages ≥ 2) that did not increase or decrease by 20% or more within 4 weeks from initial determination [4]. In the first 4-week period, all dogs were started on an RD (Royal Canin Renal Canine, Royal Canin SA, Aimargues, France; Hill's Prescription Diet Canine k/d, Hill's Pet Nutrition Inc, Topeka, Kansas, USA). 

Dogs were excluded if clinically affected or suspected to be affected by genitourinary tract inflammation or infection, cardiac disease, neoplasia, and endocrinopathies. As a standard, dogs with arterial pressure (AP) substage 3 of the IRIS staging system [12] were treated with oral amlodipine at 0.1 to 0.5 mg/kg, *q *24 hr, in order to reduce AP to substage 1 or 0 [6, 7, 13, 14]. Dogs with serum albumin concentration ≤2.0 g/dL received oral acetylsalicylic acid at 2.0 mg/kg *q *24 hr, to prevent thrombosis [6] (Shearer L, Kruth SA, Wood D. Effects of aspirin and clopidogrel on platelet function in healthydogs. J Vet Intern Med 2009; 23(3): 745 (abstract)).

### 2.2. Study Design

A randomized, blinded, placebo-controlled clinical trial was performed using a software to allocate cases (MedCalc, Version 11.3.0.0). Informed consent to participate in the study was signed by dog owners.

In the first 4 weeks following inclusion all dogs were started on an RD (Royal Canin Renal Canine, Royal Canin SA, Aimargues, France; Hill's Prescription Diet Canine k/d, Hill's Pet Nutrition Inc, Topeka, Kansas, USA). At the end of this first period, all dogs were clinically reevaluated, performing all above-mentioned laboratory and instrumental analyses and assigned to group A (RD plus placebo), or treatment group B (RD plus Renal). Compositions of the dietary supplements are provided in Table 1.

To mask the identity of the two supplements, they were formulated as powders with identical colours and contained in the same package. After assignment to group A and B, dogs were reassessed between week 4 and 8. Thereafter, examinations were scheduled every 4 months and up to 44 weeks of treatment, or earlier if worsening of clinical signs was noted by the owner.

### 2.3. Blood Sampling and Assay

During each examination, a blood sample was collected in overnight fasted dogs, and serum was obtained within 30 minutes, stored at 4°C and analyzed within 24 hours. Venous blood gas analysis (Rapidpoint 400, Bayer Health Care, Tarrytown (NY), USA) was immediately performed in all cases.

CBC and serum biochemical analysis, including albumin, total protein, glucose, bilirubin, cholesterol, amylase, alanine transferase, alkaline phosphatase, blood urea nitrogen (BUN), creatinine, ionized calcium, sodium, potassium, chloride, and phosphate, were determined by the use of standard methods (Cobas Mira, Roche Diagnostic, Basel, Switzerland). Blood samples were labelled with alphanumeric codes assigned by randomization to ensure that laboratory personals were blinded during processing. All of the above-mentioned biochemical parameters were used for inclusion and exclusion evaluation.

### 2.4. Urine Sample and Urinalysis

During abdominal ultrasonography, an echo-guided cystocentesis was performed in all dogs, by the use of a 5 mL syringe connected to a 23-gauge needle. All urine samples were put in 10 mL, sterile, evacuated collection tubes labelled with alphanumeric codes based on the previous randomization. All urine samples were analyzed by the same operator. Urines were examined within 60 minutes from collection if samples were stored at room temperature (approx. 20°C), or within 4 hours if samples were stored at 4° to 8°C. Urine sediment was obtained by centrifugation (10 minutes at 900 ×g) of 5 mL of urine, followed by removal of 4.5 mL of supernatant, and by resuspension of the remaining 0.5 mL of urine. A sample of 12 *μ*L of the resuspended urine was microscopically assessed. The supernatant was transferred into separate tubes and stored at −20°C to determine urine protein and creatinine concentration within 7 days. RBCs and WBCs were expressed as mean number of cells/10 hpf (40x magnification). Urine sediment with bacteriuria, and/or >5 RBCs or WBCs/hpf, was considered indicative of active inflammation. 

### 2.5. UPC Ratio

To calculate the UPC ratio, protein concentration (mg/dL) was measured with pyrogallol red, and creatinine (mg/dL) was measured by the use of the Jaffé method in undiluted urine that was thawed before the analysis. Analytes were measured in an automated spectrophotometer (Cobas Mira, Roche Diagnostic, Basel, Switzerland). Dogs were classified as nonproteinuric, borderline proteinuric, or proteinuric according to the IRIS staging system (UPC ratio < 0.2 = nonproteinuric, UPC ratio 0.2 to 0.5 = borderline proteinuric, and UPC ratio > 0.5 = proteinuric) [4, 12].

### 2.6. Blood Pressure Measurement

Systolic blood pressure measurements were obtained by the use of an ultrasonic Doppler device (DOP 2001, SAMED Elettromedicali srl, Merlino (LO), Italy) in all dogs [13, 14].

### 2.7. Diagnosis of Uremic Crisis

Diagnosis of uremic crisis was established by clinicians involved in patient management unaware of the supplement being administered. As previously suggested by Jacob and colleagues [1], uremic crisis was defined when the 3 following findings were observed: (i) identification of at least 2 clinical signs consistent with uremia including depression, lethargy, anorexia, vomiting, uremic breath odour; (ii) serum creatinine concentration at least 20% greater than the previously determined value; (iii) no plausible alternative for these clinical signs. 

### 2.8. Establishing Cause of Death

Causes of death were categorized as nonrenal, probably renal or renal, based on results of anamnesis, physical examination, blood and urine tests, and criteria used to define uremic crisis. To avoid bias, only dogs classified in the third category were considered to have died from a renal event (uremic crisis). Necropsies were not performed in any case.

### 2.9. Statistical Analysis

Dogs characteristics between groups were compared at the time of group assignment, and intragroup during reexamination at 4–8 weeks of treatment, using the Mann-Whitney nonparametric test. We statistically evaluated the following parameters: BCS and BW, hematocrit, serum creatinine, BUN, phosphate, blood pH, bicarbonate, and UPC ratio.

Kaplan-Meier was used to evaluate the survival probability in both groups and the Logrank test was used to compare rates of death due to uremic crisis between groups. In addition, the Kaplan-Meier was used to evaluate the probability of maintaining stable serum creatinine (serum creatinine concentration not increased above 20% compared to randomization time) in both groups, and the Logrank test was used to compare rates of creatinine variation between groups. Statistical analysis was performed with a commerciall software, using the intention-to-treat principle. Significance was defined as *P* < 0.05.

## 3. Results

### 3.1. Dogs and Groups

Thirty-one dogs were enrolled in the study. The median age of all dogs was 6 years (range 10 months–13 years). The median age of dogs in group A was 5 years. The median age of dogs in group B was 7 years. Four dogs were intact females, 16 were spayed females, 11 were males. Seven dogs were mixed breed, 2 each Dalmatian, German Shepherd, and Boxer, 1 each Beagle, Boxer, Cavalier King Charles Spaniel, American Pittbull, Dobermann, Golden Retriever, Labrador Retriever, Rottweiler, Border Collie, Bullmastiff, English Bulldog, English Cocker, English Setter, Greyhound, Irish Wolfhound, York-Shire Terrier, and Miniature Poodle.

Fifteen dogs were allocated in group A, and 16 in group B. At the time of allocation, there was no statistical difference between groups with regard to BW and BCS, hematocrit, serum creatinine, BUN, phosphate, blood pH, bicarbonate, and UPC ratio (Table 2).

Four dogs in both groups had arterial pressure (AP) in substage 3 of the IRIS staging system [12] and were treated with oral amlodipine (Norvasc, Pfizer Manufacturing Deutschland GmbH, Illertissen, Germany). Five dogs in Group A and 6 dogs in Group B had low serum albumin level (serum albumin concentration ≤ 2.0 g/dL) and received oral acetylsalicylic acid at 2.0 mg/kg *q *24 hr.

### 3.2. Followup: BW and BCS, Hematocrit, Serum Creatinine, BUN, Phosphate, Blood pH, Bicarbonate, Potassium, Calcium, and UPC Ratio

When values recorded at 4–8 weeks following supplement administration were compared with those collected at randomization time, in both groups, there was an improvement for mean serum concentrations of creatinine, BUN, phosphate, bicarbonate, and results of venous blood gas analysis, but it was statistically insignificant (Tables 3 and 4). Potassium and calcium remained stable in both groups A and B, and no episodes of hyperkalemia or of hypercalcemia were identified during the study period. There were a significant increase in BW in group B 4–8 weeks following enrolment (Table 3).

### 3.3. Associations between Dietary Supplements and Serum Creatinine or Death for Uremic Crises

**Figure 1:**
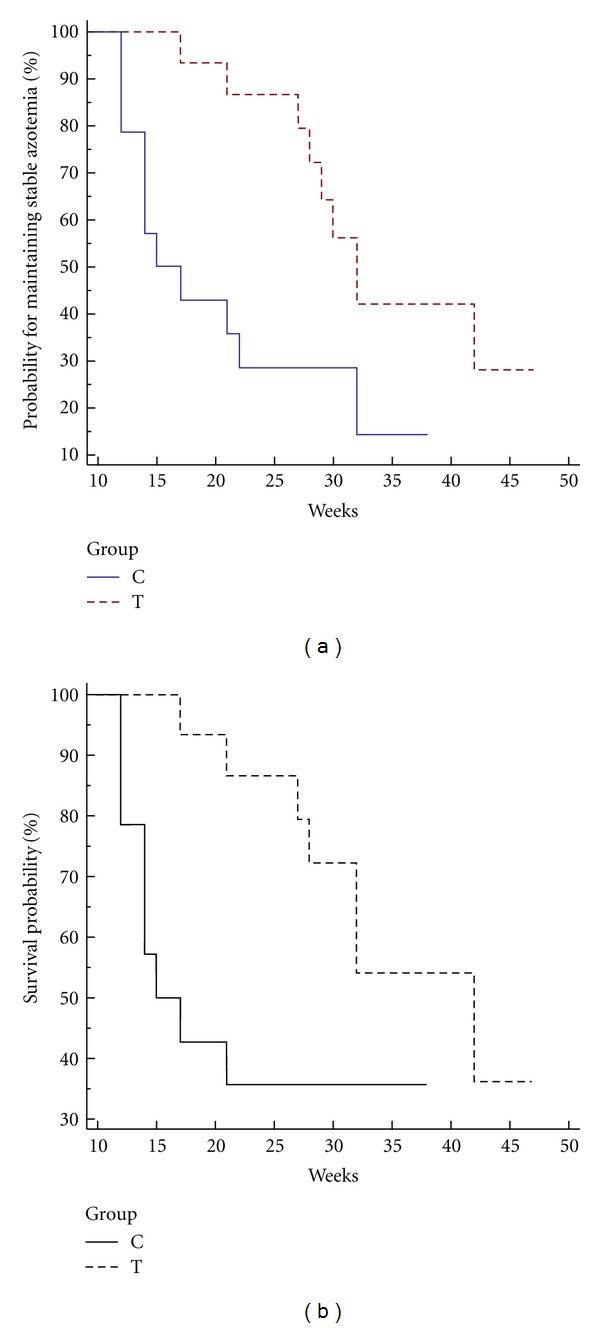
(a) Kaplan-Meier evaluating the probability of maintaining stable level of serum creatinine in treatment group B and in placebo group A. Legend: C: placebo control group A; T: treatment group B. (b) Survival curves (death from renal causes) in CKD dogs in IRIS stages 2, 3, and 4 in treatment group B and in placebo control group A. Legend: C: placebo control group A; T: treatment group B.

There was a statistically significant difference (*P* = 0.0063; chi-square = 7.44; 95% CI 0.1231 to 0.8615) between groups A and B regarding the probability of maintaining stable level of serum creatinine, with a median period of 16 weeks for group A and 32 weeks for group B (Figure 1(a)). By the end of the study, 9 out of 15 dogs in group A were dead (8 for uremic crises), against 6 out of 16 in group B (5 for uremic crises). Median survival of dogs in group B was 42 weeks and was significantly longer than in the control group, with a median survival of 16 weeks (*P* = 0.0015; chi-square = 5.88; 95% CI 0.1093 to 0.9476) (Figure 1(b)). Nonrenal causes of death included one case of aspiration pneumonia in group B, and in group A one dog died because of metastatic hemangiosarcoma. The percentage of deaths due to nonrenal causes did not differ between groups. 

## 4. Discussion

In a previously published study, admission concentration of serum creatinine did not influence survival in dogs with spontaneous CKD if an RD was administered [1]. Indeed, median survival for 21 dogs fed an RD and with a mean serum creatinine concentration of 3.3 mg/dL was 615 days, and median survival for dogs with serum creatinine between 2.0 and 3.1 mg/dL was also 615 days [1]. Differently, among 17 dogs fed a maintenance diet, median survival for dogs with a mean serum creatinine of 3.7 mg/dL was of only 252 days, whereas that of the subpopulation with serum creatinine between 2.0 and 3.1 mg/dL was 461 days [1].

In the present investigation, all dogs were fed an RD, and the mean serum creatinine concentration at the time of randomization was 5.7 mg/dL in group A and 4.9 mg/dL in group B. After 336 days of treatment, 10 out of 16 dogs in group B were alive, compared to 6 out of 15 dogs in group A, with a median survival time of 42 and 16 weeks, respectively (*P* = 0.0015), suggesting that chitosan together with alkalinizing agents are useful in maintaining long-term good clinical conditions in dogs, similar to previous studies performed in humans and cats [8–10]. In addition, the beneficial effect of the dietary supplement was evident on renal function which was stable, based on creatinine concentration, twice as much longer in group B compared to group A (*P* = 0.0063). It is worth mentioning that the improvement of blood analytes observed in the placebo-treated group A after 4–8 weeks of treatment was biased by 3 dogs that died due to severe renal failure and were therefore not included in the analysis. In contrast, none of the dogs in group B had died by 4–8 weeks and was excluded from analysis.

In humans, two possible explanations have been proposed for the reduction of serum concentrations of nitrogen metabolites, including their increased clearance due to compensatory hypertrophy of the remaining nephrons, and their enhanced excretion bound to chitosan in the digestive tract [8, 15]. Furthermore, it has been reported that chitosan can combine with acidic substances suspected to be uremic toxins, resulting in their greater excretion from the body, thus in improved clinical conditions [8, 16]; whether these same effects are exerted in dogs has not been studied but the present results may suggest that one or more of these mechanisms operate also in this species. Furthermore, because the supplement contains different substances, it cannot be excluded that phosphate binders and alkalinizing agents, or their combination, are active against uremic toxins rather than chitosan. In summary, the present study shows the beneficial effect of a commercial dietary supplement including chitosan, enteric phosphate binders, and alkalinizing agents in dogs affected by spontaneous CKD in IRIS stages 2, 3, and 4, fed an RD. The fact that serum creatinine concentration was significantly more stable and for a longer period of time in dogs receiving the supplement (Group B) is consistent with the hypothesis that delay in development of uremic crises and associated mortality rate was related, at least in part, with a reduction in the progression of renal failure.

## Figures and Tables

**Table 1:** Composition of placebo and Renal.

Placebo
Maltodextrin
Renal
Maltodextrin
Calcium carbonate (Ca 38%)
Potassium Cytrate (K 36%)
Chitosan

**Table 2:** Mean, median, and 25% and 75% for BW and BCS, hematocrit, serum creatinine, BUN, phosphate, blood pH, bicarbonate, and UPC ratio, for Group A and B at time of randomization. Differences between groups are depicted by *P* value (statistically significant *P* < 0.05).

	Group A	Group B	*P* value (A Versus B)
BW (kg)			
mean	*18.2*	*21.0*	
median	14.4	17.8	0.17
25% percentile; 75% percentile	(8.5; 29.0)	(9.7; 29.9)	
BSC (1–5)			
mean	*2.8*	*2.7*	
median	3.0	3.0	1.00
25% percentile; 75% percentile	(3.0; 3.0)	(2.0; 3.0)	
Serum Creatinine (mg/dL)			
mean	*5.7*	*4.9*	
median	3.1	4.1	0.66
25% percentile; 75% percentile	(1.9; 10.5)	(2.1; 7.23)	
BUN (mg/dL)			
mean	*97.1*	*86.2*	
median	69.0	75.1	0.76
25% percentile; 75% percentile	(32.2; 204.0)	(42.9; 150.1)	
Phosphorus (mg/dL)			
mean	*8.9*	*7.1*	
median	7.2	7.2	0.90
25% percentile; 75% percentile	(5.4; 11.0)	(5.0; 9.5)	
Blood pH			
mean	*7.30*	*7.29*	
median	7.30	7.34	0.55
25% percentile; 75% percentile	(7.25; 7.40)	(7.20; 7.39)	
HCO3^−^ (mEq/L)			
mean	*18.9*	*18.4*	
median	19.1	18.2	0.14
25% percentile; 75% percentile	(16.0; 23.4)	(14.6; 22.7)	
Hct (%)			
mean	*30.7*	*32.1*	
median	34.00	33.0	0.37
25% percentile; 75% percentile	(24.6; 39.0)	(24.5; 41.0)	
UPC ratio			
mean	*2.90*	*0.62*	
median	0.68	0.39	0.41
25% percentile; 75% percentile	(0.24; 1.60)	(0.29; 0.80)	

**Table 3:** Mean, median, and 25% and 75% percentile for BW and BCS, hematocrit, serum creatinine, BUN, phosphate, blood pH, bicarbonate, and UPC ratio, for group B at time of randomization (T0) and after 4–8 weeks (T1) of treatment. Differences between values at different examinations are depicted by *P* value (statistically significant *P* < 0.05).

	T0	T1	*P* value (T0 Versus T1)
BW (kg)			
mean	*21.0*	*23.9*	
median	17.8	23.0	0.06
25% percentile; 75% percentile	(9.7; 29.9)	(9.5; 33.9)	
BSC (1–5)			
mean	*2.7*	*2.8*	
median	3.0	3.0	1.00
25% percentile; 75% percentile	(2.0; 3.0)	(2.5; 3.0)	
Serum Creatinine (mg/dL)			
mean	*4.9*	*4.4*	
median	4.1	3.4	0.50
25% percentile; 75% percentile	(2.1; 7.23)	(1.7; 6.0)	
BUN (mg/dL)			
mean	*86.2*	*87.6*	
median	75.1	63.3	0.55
25% percentile; 75% percentile	(42.9; 150.1)	(55.0; 77.0)	
Phosphorus (mg/dL)			
mean	*7.1*	*6.9*	
median	7.2	5.4	0.87
25% percentile; 75% percentile	(5.0; 9.5)	(4.2; 10.7)	
Blood pH			
mean	*7.29*	*7.30*	
median	7.34	7.30	0.69
25% percentile; 75% percentile	(7.20; 7.39)	(7.25; 7.40)	
HCO3^−^ (mEq/L)			
mean	*18.4*	*19.8*	
median	18.2	20.5	0.20
25% percentile; 75% percentile	(14.6; 22.7)	(16.1; 22.6)	
Hct (%)			
mean	*32.1*	*34.2*	
median	33.0	34.0	0.49
25% percentile; 75% percentile	(24.5; 41.0)	(29.6; 40.7)	
UPC ratio			
mean	*0.62*	*0.58*	
median	0.39	0.3	0.39
25% percentile; 75% percentile	(0.29; 0.80)	(0.25; 0.79)	

**Table 4:** Mean, median, 25% and 75% percentile for BW and BCS, hematocrit, serum creatinine, BUN, phosphate, blood pH, bicarbonate, and UPC ratio, for group A at time of randomization (T0) and after 4–8 weeks (T1) of treatment. Difference between values at different examinations are depicted by *P*-value (statistically significant *P* < 0.05).

	T0	T1	*P* value (T0 Versus T1)
BW (kg)			
mean	*18.2*	*24.4*	0.66
median	14.4	27.9	
25% percentile; 75% percentile	(8.5; 29.0)	(18.1; 31.9)	
BSC (1–5)			
mean	*2.8*	*3.2*	1.00
median	3.0	3.0	
25% percentile; 75% percentile	(3.0; 3.0)	(3.0; 4.0)	
Serum Creatinine (mg/dL)			
mean	*5.7*	*4.2*	0.77
median	3.1	2.0	
25% percentile; 75% percentile	(1.9; 10.5)	(1.5; 5.8)	
BUN (mg/dL)			
mean	*97.1*	*77.0*	
median	69.0	32.8	0.54
25% percentile; 75% percentile	(32.2; 204.0)	(18.9; 121.6)	
Phosphorus (mg/dL)			
mean	*8.9*	*6.6*	
median	7.2	5.5	0.97
25% percentile; 75% percentile	(5.4; 11.0)	(4.7; 7.8)	
Blood pH			
mean	*7.30*	*7.32*	
median	7.30	7.30	0.65
25% percentile; 75% percentile	(7.25; 7.40)	(7.30; 7.35)	
HCO3^−^ (mEq/L)			
mean	*18.9*	*20.2*	
median	19.1	19.5	0.43
25% percentile; 75% percentile	(16.0; 23.4)	(16.3; 24.4)	
Hct (%)			
mean	*30.7*	*38.7*	
median	34.0	40.0	0.40
25% percentile; 75% percentile	(24.6; 39.0)	(28.8; 45.3)	
UPC ratio			
mean	*2.90*	*1.39*	
median	0.68	0.55	0.54
25% percentile; 75% percentile	(0.24; 1.60)	(0.26; 0.70)	

## Abbreviations

CKD: Chronic kidney disease
RD: Renal diet
IRIS: International Renal Interest Society
BW: Body weight
BCS: Body condition score
BUN: Blood urea nitrogen
UPC: Urine protein–to-creatinine
AP: Arterial pressure
RR: Relative risk
95% CI: 95% confidence interval.
